# Differential effect of ubiquitous and germline depletion of Integrator complex function on *C. elegans* physiology

**DOI:** 10.1242/bio.061930

**Published:** 2025-04-10

**Authors:** Brandon M. Waddell, Alice R. Roy, Carlos Z. Verdugo, Cheng-Wei Wu

**Affiliations:** ^1^ Department of Veterinary Biomedical Sciences, Western College of Veterinary Medicine, University of Saskatchewan, Saskatoon, SK S7N 5B4, Canada; ^2^ Toxicology Centre, University of Saskatchewan, Saskatoon, SK S7N 5B3, Canada; ^3^ Department of Biochemistry, Microbiology and Immunology, College of Medicine, University of Saskatchewan, Saskatoon, SK S7N 5E5, Canada

**Keywords:** Integrator, SnRNA, *C. elegans*, Development, Aging

## Abstract

The Integrator is a metazoan-conserved protein complex with endonuclease activity that functions to cleave various RNA substrates to shape transcriptome homeostasis by coordinating small nuclear RNA biogenesis to premature transcription termination. Depletion of Integrator results in developmental defects across different model systems and has emerged as a causative factor in human neurodevelopmental syndromes. Here, we used the model system *Caenorhabditis elegans* to enable study of the temporal effects of Integrator depletion on various physiological parameters with the auxin-inducible degron system that permitted depletion of INTS-4 (Integrator subunit) catalytic subunit of the protein complex. We found that Integrator activity is critical and required for *C. elegans* development within the L1 larval stage but becomes dispensable for development and lifespan after the animals have reached the L2/L3 stage. Depletion of INTS-4 only shortened lifespan if auxin was introduced at the L1 stage, suggesting that the previously described lifespan reduction by Integrator inhibition is linked to developmental growth defects. We also found that while germline-specific degradation of Integrator results in the accumulation of misprocessed snRNA transcript, it did not impair the development or lifespan but surprisingly increased progeny production. Together, our study illustrates a temporal, and a potentially tissue-specific requirement of the Integrator complex function in shaping whole organism development, aging, and reproduction.

## Teaser

**Summary:** Integrator has emerged as a critical protein complex for metazoan development. In this study, we used the auxin–degron system to explore tissue and temporal requirement of the Integrator in *C. elegans* development and aging.

## INTRODUCTION

The Integrator is a conserved metazoan protein complex that was discovered in 2005 as the mysterious protein machinery that catalyzes the 3′ cleavage and subsequent maturation of snRNA (small nuclear RNA) transcripts post-transcription (Baillat et al., 2005). The non-coding snRNA transcripts are unique in that after transcription by RNA polymerase, a 3′ endolytic cleavage event is required to produce a shortened and mature snRNA transcript that is then incorporated into snRNPs (small nuclear ribonucleoprotein) as part of the spliceosome to regulate RNA splicing (Baillat and Wagner, 2015; Wilkinson et al., 2020). Since its initial discovery, the Integrator has also been found to exhibit cleavage activity towards other RNA substrates including protein-coding genes during transcriptional pause-release and piRNAs (piwi-interacting RNAs) for transposon silencing to broadly contribute to transcriptome regulation and stability (Beltran et al., 2021; Gardini et al., 2014).

Over the past decade, studies in metazoan models have revealed that the Integrator function is critical in many aspects of development. Initial studies in *Drosophila* revealed that mutations to subunits 4 and 7 resulted in elevated levels of misprocessed snRNA transcripts and are associated with larval arrest at the second and third instar developmental stages (Ezzeddine et al., 2010; Rutkowski and Warren, 2009). Zebrafish with mutation to subunit 6 induced by ENU mutagenesis exhibit a dorsalized phenotype characterized by delayed epiboly initiation and a high post-fertilization mortality rate (Kapp et al., 2013). In *C. elegans,* the depletion of multiple Integrator subunits results in a larval arrest, with the most severe phenotype observed when INTS-4 encoding the catalytic subunit is knocked down (Gómez-Orte et al., 2019; Wu et al., 2019). Defects to Integrator have also emerged as a causative factor for various human pathology, with mutations to subunits 1, 8, and 11 linked to severe neurodevelopmental syndromes characterized by intellectual disability and motor impairments (Oegema et al., 2017; Tepe et al., 2023; Zhang et al., 2020).

Recently, we identified in the model *C. elegans* a role for the *csr-1* (Chromosome-Segregation and RNAi deficient) gene encoding an Argonaute protein in regulating snRNA 3′ processing by controlling the expression of INTS-4 protein in the germline (Waddell and Wu, 2024). Interestingly, we reported that loss of *csr-1* specifically disrupts INTS-4 expression in the germline, suggesting tissue-specific regulators of Integrator function. In this study, we utilized the auxin-inducible degradation (AID) system to construct *C. elegans* strains that allow for rapid ubiquitous or germline-specific inactivation of the Integrator complex activity via INTS-4 degradation to determine its effect on *C. elegans* developmental, reproductive, and aging physiology. The use insertion of the AID epitope tag to endogenous gene loci in combination with the expression of the F-box protein TIR1 has enabled rapid and reversible degradation of the target protein in the presence of the auxin pheromone (Ashley et al., 2021; Zhang et al., 2015). Our study highlights how this AID system can enable rapid INTS-4 degradation by controlling the timing of auxin exposure and the co-expression of TIR1 under different promoters to dissect the requirement of the Integrator complex in whole organism development in a tissue and temporal-specific manner.

## RESULTS AND DISCUSSION

### Germline-specific degradation of INTS-4

**Fig. 1.**
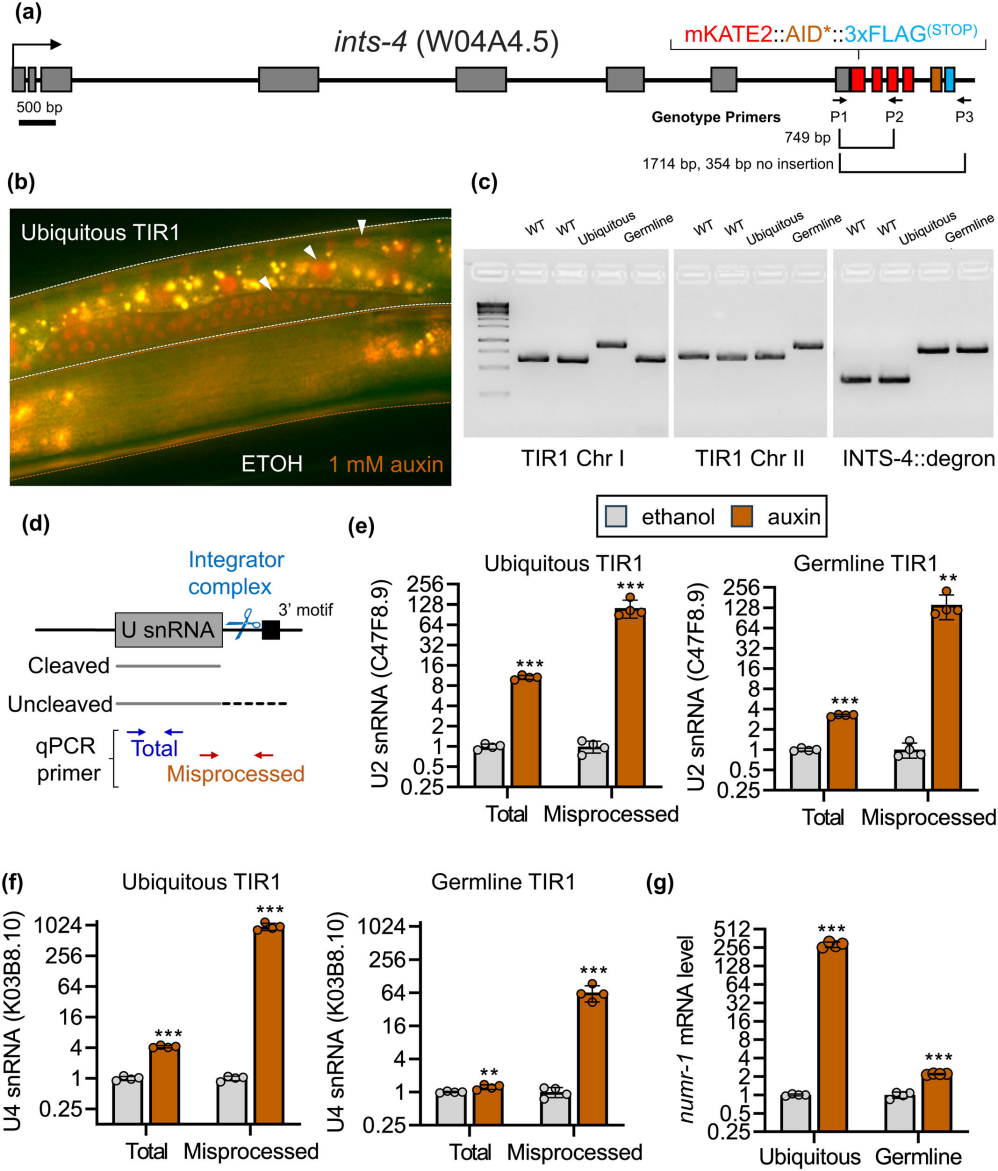
**Ubiquitous and germline depletion of INTS-4.** (A) Schematic for mKATE2::AID*::3xFLAG knock-in before the stop codon of the *ints-4* locus (referred to as INTS-4::degron). The positions of the primers used to PCR genotype the knock-in are shown. (B) Representative fluorescent micrograph showing effects of ethanol or 1 mM auxin exposure on expression of INTS-4 co-expressing the ubiquitous TIR1 driven by the *eft-3* promoter. Top worm outlined in white was treated with the ethanol as a control solvent and the bottom worm outlined in brown was treated with 1 mM of auxin. Arrowhead indicates mKate2 expression in the hypodermis, intestine, and germline of the control ethanol treated worm. The mKate2 signals were lost upon auxin exposure. (C) Agarose gel showing PCR genotyping of single copy TIR1 insertion and INTS-4::degron knock-in of wildtype N2 (WT) and strains expressing INTS-4::degron with ubiquitous TIR1 (knock-in on chromosome 1) or germline TIR1 (knock-in on chromosome 2). Primer positions to genotype INTS-4::degron are shown in (A) and primers used for the TIR1 genotype were previously described by (Ashley et al., 2021). GeneRuler 1 kb ladder is shown with the bottom 4 markers indicate 1000, 750, 500, and 250 bp. (D) Illustration of U snRNA processing by the Integrator complex and qPCR primer design to differentiate the expression of total and misprocessed snRNA transcripts. Relative expression of total and misprocessed (E) U2 snRNA and (F) U4 snRNA in worm strains expressing ubiquitous or germline TIR1 exposed to ethanol (control) or 1 mM of auxin. (G) Relative expression of *numr-1* transcript in worm strains expression ubiquitous or germline TIR1 exposed to ethanol (control) or 1 mM of auxin. Bar graphs indicate mean±s.d. with individual data points (*N*=4) shown. **P<0.01, ***P<0.001 as determined by multiple *t*-tests corrected with the Holm-Sidak method.

We previously reported through a genetic screen that the Argonaute encoding *csr-1* gene influences snRNA processing by regulating INTS-4 expression in the *C. elegans* germline (Waddell and Wu, 2024). To explore how germline-specific degradation of INTS-4 relates to ubiquitous depletion, we introduced the F-box protein TIR1 driven by the *sun-1* promoter to our previously constructed *C. elegans* strain expressing the mKate2::AID*::3xFLAG tag inserted at the C-terminal end of the *ints-4* loci (Waddell and Wu, 2024) (Fig. 1A-C). This combination permits germline-specific degradation of INTS-4 in the presence of auxin, facilitated by the expression of TIR1 expressed under the *sun-1* promoter, as compared to ubiquitous depletion achieved by the expression of TIR1 driven by the *eft-3* promoter. When TIR1 is ubiquitously expressed, exposure to auxin (Fig. 1B; bottom worm, outlined in brown) caused the loss of INTS-4 mKate2 fluorescence that is observed in the control worm treated with only the ethanol solvent (Fig. 1B; top worm, outlined in white).

To determine the effect of germline-specific depletion of INTS-4 on snRNA 3′ processing relative to ubiquitous depletion, we use a combination of DNA primers that specifically detect the misprocessed form of the snRNA transcript caused by transcriptional read-through as a proxy for Integrator malfunction (Fig. 1D) (Gómez-Orte et al., 2019; Skaar et al., 2015; Waddell and Wu, 2024). Depletion of ubiquitous and germline INTS-4 both led to moderate increases in total levels of U2 snRNA (C47F8.9) but strongly increased misprocessed transcript levels (Fig. 1E). We then measured another snRNA transcript (U4, K03E8.10) and found a similar trend where the total U4 levels were slightly upregulated and the misprocessed transcript was strongly elevated (Fig. 1F). Interestingly, the degree of U2 snRNA misprocessing was comparable between the two strains, whereas U4 snRNA showed a ∼15-fold increase in misprocessing in the ubiquitous INTS-4 depletion vs germline only (ubiquitous: 957±76.68 versus germline: 64.39±10.49-fold change). This indicates that specific transcripts coding snRNA genes may be differentially expressed in distinct *C. elegans* tissues, where C47F8.9 is highly expressed in the germline and K03E8.10 is ubiquitously expressed. This may in part explain why snRNA genes are encoded by multiple transcripts in *C. elegans* to potentially allow for broad expression of each U class contributed by tissue-specific expression of distinct transcripts.

We also measured the expression of *numr-1*, which encodes a nucleolar stress responsive gene that we previously showed to be highly activated upon Integrator disruption (Hong et al., 2024; Wu et al., 2019). As expected, ubiquitous depletion of INTS-4 strongly activated the expression of *numr-1*, however, germline degradation of INTS-4 only led to a slight but statistically significant increase in *numr-1* by ∼2.2-fold (Fig. 1G). The lack of *numr-1* activation in response to germline degradation is unsurprising as *numr-1* functions primarily in the *C. elegans* intestine, for which Integrator activity in this tissue would be unaffected by germline-specific INTS-4 depletion (Tvermoes et al., 2010; Wu et al., 2019). Overall, the results here indicate that depletion of germline INTS-4 alone can result in significant misprocessing of snRNA transcripts to indicate evidence of Integrator malfunction.

### Tissue and temporal requirement of INTS-4 for development

**Fig. 2.**
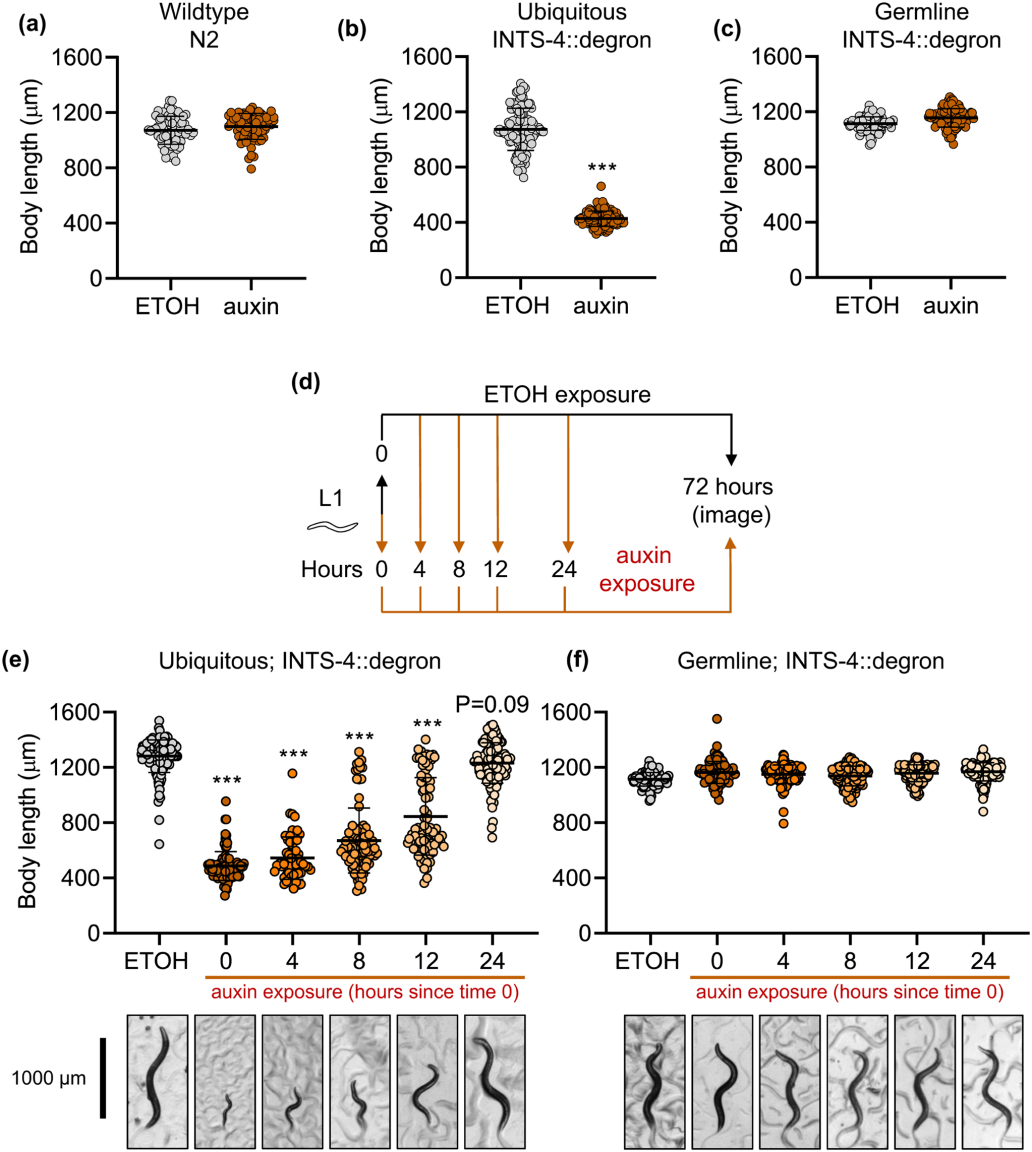
**Developmental requirement of ubiquitous and germline INTS-4.** Development of *C. elegans* as determined by body length in (A) N2, (B) INTS-4::degron strain expressing ubiquitous TIR1, and (C) INTS-4::degron expressing germline TIR after exposure to ethanol or 1 mM auxin starting at L1 followed by body size measurement after 72 h. ***P<0.001 as determined by Student's *t*-test. (D) Workflow to analyze the effect of delayed auxin exposure during development on body growth. Effect of delayed auxin exposure on body size growth of INTS-4::degron strain expressing (E) ubiquitous or (F) germline TIR1. ***P<0.001 as determined by one-way ANOVA test. All data shown includes two independent trials with each condition containing *N*=68-72 worms in (A), *N*=126-128 worms in (B), *N*=102-116 worms in (C), *N*=62-138 in (E) and *N*=101-116 worms in (F).

Mutations to genes encoding subunits of the Integrator complex have recently emerged as a causative factor in various forms of neurodevelopment syndromes in humans (Oegema et al., 2017; Zhang et al., 2020). In the *C. elegans* model, a hallmark of Integrator disruption through systemic RNAi silencing is the arrest of larval development, with the most severe phenotype seen when the catalytic subunit-4 is depleted (Gómez-Orte et al., 2019; Wu et al., 2019). We next sought to determine the effect of ubiquitous or germline depletion of INTS-4 on larval development as accomplished by the auxin-degron system. To account for any non-specific effects stemming from auxin exposure, we first tested its effect on the N2 wildtype worms for which we observed no difference in worm body size after 72 h of growth from L1 (Fig. 2A). Next, we found that auxin exposure initiated at the L1 stage to INTS-4::degron worms with ubiquitous TIR1 expression resulted in a 60% decrease in body size compared to the ethanol control, which is similar to the results previously observed with *ints-4* RNAi (Gómez-Orte et al., 2019; Wu et al., 2019) (Fig. 2B). When auxin was exposed to L1 INTS-4::degron worms expressing TIR1 in the germline, there was no difference in body growth compared to ethanol control (Fig. 2C). This suggested that while depletion of germline INTS-4 impaired Integrator activity as evidenced by the increase in snRNA misprocessing (Fig. 1E-F), this deficiency localized to the germline does not impair whole animal development of *C. elegans*.

Given the flexibility of the AID system that enables rapid degradation of the target protein by controlling the timing of auxin exposure, we were next interested in determining whether there is a specific window for which ubiquitous INTS-4 is required for *C. elegans* development. We delayed auxin exposure by 0, 4, 8, 12, and 24-h from the synchronized L1 stage followed by measuring the worm body size at the 72-h mark; a corresponding control that was continuously grown on ethanol only condition was prepared in parallel. (Fig. 2D). We found that auxin exposure within the first 12 h resulted in larval arrest and a significant reduction in body size of *C. elegans* expressing ubiquitous TIR1 compared to worms continuously grown on ethanol control plates (Fig. 2E). Increasing the delay exposure timing resulted in the progressive increase in body size suggesting the duration for which INTS-4 is maintained is correlated to body size growth (i.e. 4 h: *x̄*=544 µm, 8 h: *x̄*=671 µm, 12 h: *x̄*=845 µm). Interestingly, worms that were allowed to develop normally for 24 h on ethanol plates followed by auxin exposure did not experience a significant reduction in body size (Fig. 2E). We also repeated this delayed auxin exposure experiment in the worm strain expressing germline TIR1 and saw no effect on body growth regardless of when auxin was introduced during development (Fig. 2F), confirming the results we show in Fig. 2C.

Overall, these results provide new evidence to support a temporal requirement of the Integrator complex for *C. elegans* development and suggest that Integrator activity is critical during the L1 larval stage and becomes nonessential for body growth as the worm enters the L2/L3 stage of development. Given that alternative splicing has been shown to play an important role in *C. elegans* development, it is possible that the depletion of Integrator in L1 may contribute to growth arrest through disruption to RNA splicing caused by the loss of snRNA processing required for functional spliceosome assembly (Barberan-Soler and Zahler, 2008; Ramani et al., 2011).

### Germline INTS-4 influence on reproduction

**Fig. 3.**
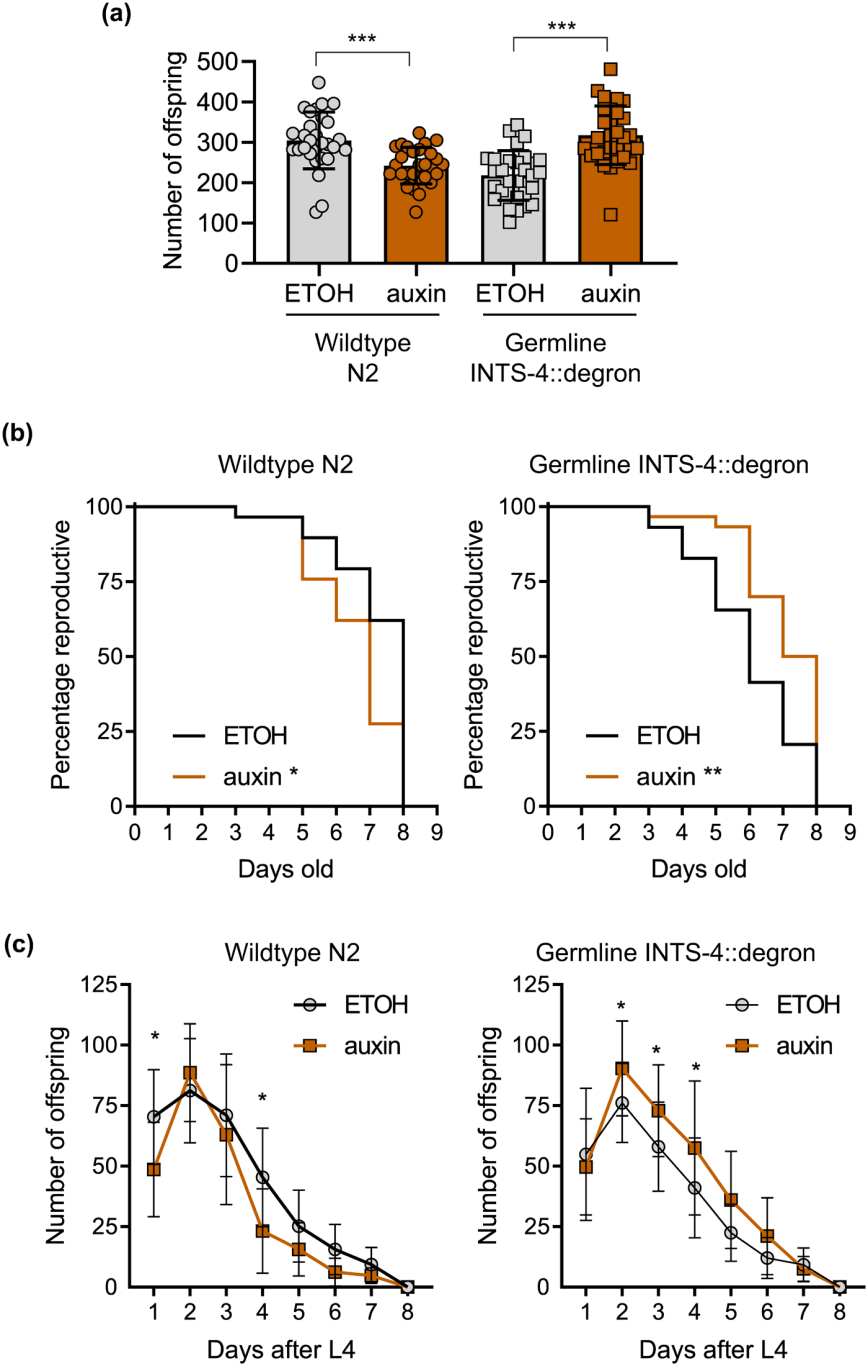
**Germline INTS-4 depletion alters reproduction.** (A) Total offspring number, (B) reproductive span, and (C) daily offspring number of wildtype N2 and INTS-4::degron strain expressing germline TIR1 after exposure to ethanol or 1 mM auxin starting from L1. Data points were obtained from *N*=29-30 worms per condition combined from three independent trials. One-way ANOVA was used for the statistical test in (A) and (C) with the log-rank test used for (B). *P<0.05, **P<0.01, ***P<0.001 as compared to the ETOH control.

Recently, Integrator was shown to be required to promote ovarian germ cell differentiation in *Drosophila*, which may suggest a regulatory role for this protein complex within the germline in controlling development but under different contexts (Liu et al., 2023). Given that germ cells do not differentiate into sperm or oocyte until the L3/L4 stage, we questioned whether depletion of germline Integrator function would alter *C. elegans* reproductive physiology rather than larval development (Pazdernik and Schedl, 2013). We next determined the effects of INTS-4 germline depletion on *C. elegans* reproduction to determine potential effects on germline function. We found that auxin treatment caused a slight decrease in the N2 wildtype brood size, which was similarly observed in a previous study (Fig. 3A) (Zhang et al., 2015). Unexpectedly, we found that auxin exposure in INTS-4::degron strain expressing germline TIR1 led to a significant increase in brood size (Fig. 3A). We then analyzed the daily reproduction data and found that while depletion of germline INTS-4 did not extend the overall reproduction window, there was a significant increase in mean reproductive span after auxin treatment compared to ethanol (auxin: 7.07±0.22 days versus ETOH: 6.03±0.28 days), an effect that was not observed in the N2 wildtype (Fig. 3B). Correspondingly, we observed that germline INTS-4 depletion resulted in a significant increase in offspring produced from days 2-4, which typically represents the peak of the *C. elegans* reproductive window (Kocsisova et al., 2019) (Fig. 3C).

While it is unclear why depletion of germline INTS-4 would enhance reproduction, it is possible that this phenotype may be linked to the pause-release function of the Integrator in regulating protein-coding genes (Gardini et al., 2014). In this context, the inactivation of the Integrator has been shown to result in the upregulation of genes that would normally be prematurely terminated during transcriptional pausing via cleavage by the Integrator (Gardini et al., 2014; Tatomer et al., 2019). We analyzed previously published RNA-seq data and found that the knockdown of *ints-4* led to a broad upregulation of several classes of genes involved in spermatogenesis and embryogenesis. These include *fer-1* (FERtilization defective), 2 *gsp* (Glc Seven like Phosphatase) class genes, 3 *spch* (SPerm CHromatin enriched) class genes, and 16 *spe* (defective SPErmatogenesis) class genes (Fig. S1). We speculate that the increases in these genes may serve a possible mechanism through which *ints-4* depletion altered germline functions to increase brood size (Argon and Ward, 1980; Billmyre et al., 2019; Gómez-Orte et al., 2019; Nishimura and L'Hernault, 2010; Samson et al., 2014). However, we note the limitation in our study in that we did not use qPCR to directly measure the changes to these gene expression.

### Contribution of INTS-4 to lifespan

**Fig. 4.**
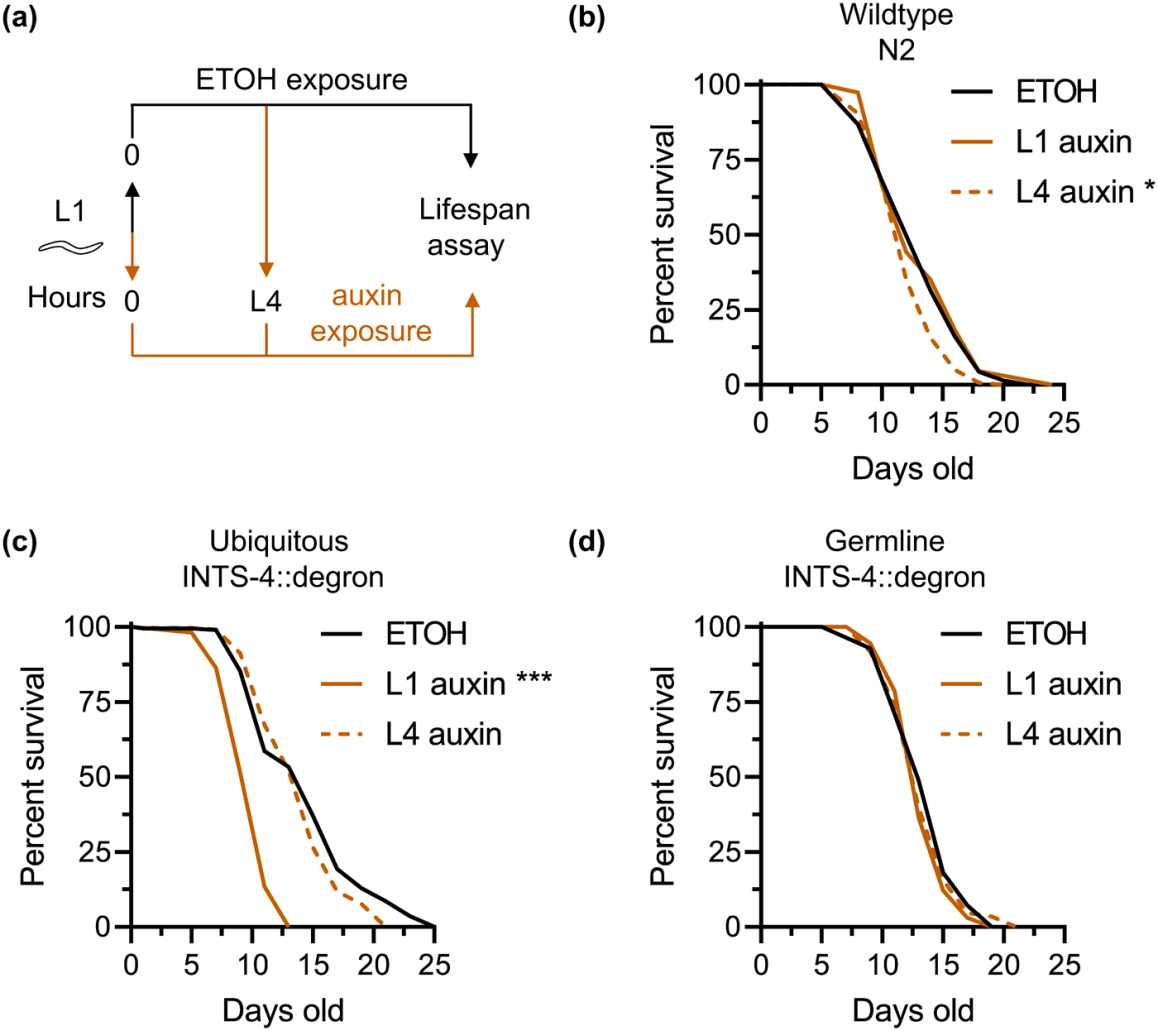
**Impact of ubiquitous and germline INTS-4 depletion on lifespan.** (A) Workflow to initiate ubiquitous or germline INTS-4 degradation from the L1 or L4 stage to determine its impact on lifespan. Lifespan analysis of (B) N2, (C) INTS-4::degron strain expressing ubiquitous TIR1, and (D) INTS-4::degron expressing germline TIR1 after exposure to ethanol or 1 mM auxin starting at L1 or L4. Three independent trials were performed for each lifespan assay with complete statistics presented in Table S1. Trial 2 is shown for all strains. *P<0.05, ***P<0.001 as determined by the log-rank test.

We previously showed that RNAi knockdown of *ints-4* results in a significant reduction in *C. elegans* lifespan, implicating a possible role for the Integrator complex in aging (Wu et al., 2019). Integrator malfunction in contributing to aging was also recently demonstrated in *Drosophila* where expression of loss of function variant of INTS-11 shortened the fruit fly lifespan (Tepe et al., 2023). To determine whether the reduced lifespan previously observed after *ints-4* knockdown via RNAi was related to its developmental defect, we compared the effect on lifespan if INTS-4 was depleted via auxin exposure starting from L1 or at the final L4 larval stage (Fig. 4A). We implemented the auxin exposure to N2 wildtype worms to account for any baseline effects and found that while L1 exposure did not influence lifespan, L4 exposure led to a small but reproducible reduction of lifespan by an average of 8% (Fig. 4B, Table S1). As expected, L1 depletion of INTS-4 in worms expressing ubiquitous TIR1 resulted in larval arrest and these worms showed an average reduction in lifespan by 33% compared to ethanol-exposed worms that were able to fully develop into adults (Fig. 4C). Interestingly, when INTS-4 was depleted ubiquitously starting from the L4 stage to bypass any potential developmental defects, there was no significant change to lifespan (Fig. 4C). This suggests that the shortened lifespan previously observed from *ints-4* RNAi where knockdown was introduced at the L1 stage may be pleiotropically linked with the developmental defect phenotype. When Integrator activity was depleted in worms that had reached near full adulthood, there was no negative effect on lifespan (Fig. 4C). We also repeated this experiment to test for the effect of L1 or L4 depletion of germline INTS-4 on lifespan. We observed that auxin-induced degradation of INTS-4 starting at either the L1 or L4 stage had no effect on *C. elegans* lifespan, suggesting that germline Integrator function does not influence aging.

The lifespan data show that depletion of ubiquitous INTS-4 only shortened lifespan if it occurred during larval development. The ubiquitous TIR1 expression is driven by the *eft-3* promoter that is strongly expressed in somatic cells, where their mitotic division occurs during early embryonic and larval stages of development and cells become irreversibly arrested in a postmitotic state once they reach adulthood (Kipreos and van den Heuvel, 2019). In this context, Integrator functions may only be critical in actively dividing cells during larval development and becomes dispensable once cells have fully differentiated. This may reflect why the loss of Integrator during the larval stage led to a shortened lifespan that may be linked to the developmental defect, while having no effect when the depletion occurs during adulthood. Given that the *eft-3* promoter is widely expressed across all major tissues of the worm, future studies using different gene promoter to drive tissue specific TIR1 expression will permit a deeper investigation into identifying if a specific tissue or cell type is responsible for the larval arrest and shortened lifespan observed when ubiquitous INTS-4 is depleted during development. In conclusion, our data here contribute new insights into temporal specific requirement of Integrator function in *C. elegans* developmental physiology and provide an initial insight into how the auxin-degron toolset may permit future investigation of the Integrator complex's contribution to metazoan development in a tissue and developmental-specific context.

## MATERIALS AND METHODS

### *C. elegans* maintenance

This study used the following strains: N2 Bristol wildtype, JDW10 wrdSi3 [sun-1p::TIR1::F2A::mTagBFP2::AID*::NLS::tbb-2 3′UTR] (II:0.77); JDW225 wrdSi23 [eft-3p::TIR1::F2A::mTagBFP2::AID*::NLS::tbb-2 3′UTR] (I:-5.32), MWU172 cwwSi1[ints-4::mKATE2::AID*::3xFLAG], MWU193 cwwSi1[ints-4::mKATE2::AID*::3xFLAG]; wrdSi23 [eft-3p::TIR1::F2A::mTagBFP2::AID*::NLS::tbb-2 3′UTR] (I:-5.32), MWU212 cwwSi1[ints-4::mKATE2::AID*::3xFLAG]; wrdSi3 [sun-1p::TIR1::F2A::mTagBFP2::AID*::NLS::tbb-2 3′UTR] (II:0.77). MWU193 was generated by crossing MWU172 with JDW225 and MWU212 was generated by crossing MWU172 with JDW10. All strains were cultured on nematode growth medium (NGM) agar plates using standard conditions as described by Brenner (1974). For auxin experiments, a stock of 400 mM 3-Indoleacetic acid (Millipore Sigma, I2886) was prepared in 100% ethanol and used to make an NGM agar auxin plate containing a final concentration of 1 mM. A corresponding control NGM plate containing 0.25% ethanol was prepared. The standard bacteria *E. coli* OP50 was seeded on the NGM agar plates as a food source.

### PCR genotyping

A total of 24 single *F*_2_ progeny from a mating cross experiment were individually isolated and allowed to self-reproduce for 1 day followed by single worm lysis to genotype for endogenous *ints-4* edit created via CRISPR/Cas9, and for knock-in of TIR1 driven by ubiquitous or germline promoters (Ashley et al., 2021; Dickinson et al., 2015). The LongAmp Taq polymerase (NEB, M0323S) was used for DNA amplification and the DNA primers used to genotype *ints-4* insertion were as follows: P1: 5′-AGTAACATGCTCATCCCCGT-3′, P2: 5′-GTCCCTCAAGTCCTCCGTC-3′, and P3: 5′-ACTGTCAATTTGCCGAACACT-3′. Primers used to genotype TIR1 insertion were previously described by (Ashley et al., 2021). Population homozygous for *ints-4* edit and the desired TIR1 insertion were re-confirmed through genomic DNA extraction followed by PCR genotyping.

### RNA extraction and qPCR

Methods used for RNA extraction and cDNA library construction were as previously described (Waddell and Wu, 2024). Briefly, age-synchronized N2, MWU193, or MWU212 worms at the L1 stage were grown on an NGM agar plate containing 0.25% ethanol (control) or 1 mM auxin for 72 h followed by RNA extraction using the Purelink RNA mini kit (ThermoFisher, 12183020) with lysis obtained by sonication with a QSonica Q55 sonicator. RNA was normalized to a final concentration of 200 ng/µl and treated with DNAseI (ThermoFisher, EN0521) followed by cDNA library synthesis with random priming using the Invitrogen Mutiscribe reverse transcriptase (ThermoFisher, 4311235). The PowerUp SYBR Green Mastermix (ThermoFisher, A25741) was used to measure the expression of total and misprocessed U2 and U4 snRNA transcript and the *numr-1* gene. Relative gene expression was normalized to the housekeeping gene *cdc42*. Primers used for this experiment were previously described in (Waddell and Wu, 2024).

### Fluorescent microscopy

To capture fluorescent images of endogenous INTS-4 tagged to mKate2, synchronized MWU193 L4 worms were moved to NGM agar plates containing ethanol or 1 mM of auxin for 2 h to induce INTS-4 degradation followed by mounting on a glass slide containing 2% agarose and 0.65% sodium azide to immobilize the worms. The DeltaVision (GE) system was used to capture the mKate2 fluorescent signals through the TRITC filter with an additional image taken with the DAPI filter to identify non-specific signals emitted from the intestinal cells. ImageJ was used to colourize and merge the greyscale images captured by the TRITC and DAPI filters to create the composite image.

### *C. elegans* physiological assays

For lifespan assays, synchronized L1 N2, MWU193, or MWU212 worms were grown on NGM agar containing 0.25% ethanol or 1 mM auxin seeded with *E. coli* OP50 and maintained at 25°C. For L4 exposure, L1 worms were first grown on NGM agar plates with 0.25% ethanol followed by transfer to NGM agar plates containing 1 mM auxin. Worms were scored every 2 days for death via gentle prodding with a sterilized platinum pick. Worms were considered dead if they did not respond to the touch and were censored if they demonstrated a protruding vulva or gonad. Manual picking was performed to segregate the worms from their progeny daily during the reproductive window to avoid mixing of P_0_ population with the offspring. Three independent lifespan trials were performed for all strains with complete statistics shown in Table S1. Lifespan assays were performed at 25°C as we previously reported high incidences of censorship when performing lifespan assays with MWU193 at 20°C (Waddell and Wu, 2024).

For developmental assays, synchronized L1 N2, MWU193, or MWU212 worms were grown on NGM agar containing 0.25% ethanol or 1 mM auxin seeded with *E. coli* OP50 and maintained at 20°C for 72 h followed by imaging with an Olympus SZX61 fitted with a Retiga R3 microscope camera (Murray et al., 2020). For delayed auxin exposure, L1 worms were first grown on 0.25% ethanol NGM agar followed by transfer to 1 mM auxin NGM agar plates after 0, 4, 8, 12, or 24 h and maintained until a total of 72 h had passed since time 0 for imaging. Body size measurements were performed using the measure function in ImageJ to determine the worm's body size. Two independent development assay trials were performed with the number of worms measured in each condition indicated in the figure legends.

For the reproductive assay, synchronized L1 N2, MWU193, or MWU212 worms were grown on NGM agar containing 0.25% ethanol or 1 mM auxin seeded with *E. coli* OP50 and maintained at 20°C until the worms reached L4. Individual L4 worms were picked into a single OP50 seeded NGM agar plates containing 0.25% ethanol or 1 mM auxin. The number of eggs and hatched progeny were counted daily and each worm was moved to a new plate daily. This process was repeated for 8 days until reproduction has ceased. The total number of eggs and hatched progeny were counted to obtain the total brood size. Three independent assays were performed with nine to ten worms measured per condition in each trial.

### Statistical analyses

GraphPad Prism 8.4.3 was used to generate the graphs and perform statistical analyses. *t*-test was used for comparison of two groups and corrected for multiple tests using the Holm-Sidak method when applicable. For the comparison of more than two groups, one-way ANOVA with the Dunnett test was used. OASIS2 was used to determine statistics for the lifespan assays and significance was tested using the log-rank test (Han et al., 2016). Statistical significance was indicated as: *P≤0.05, **P≤0.01, ***P≤0.001.

## Supplementary Material

10.1242/biolopen.061930_sup1 Supplementary information

Table S1. Lifespan assay statistics
