# Supplementary material for: Differential effect of ubiquitous and germline depletion of Integrator complex function on C. elegans physiology
Source: Biol Open. 2025 Apr 10;14(4):bio061930. doi: 10.1242/bio.061930 (PMC12010912; doi:10.1242/bio.061930)
Supplement: Supplementary information [file biolopen-14-061930-s1.pdf]

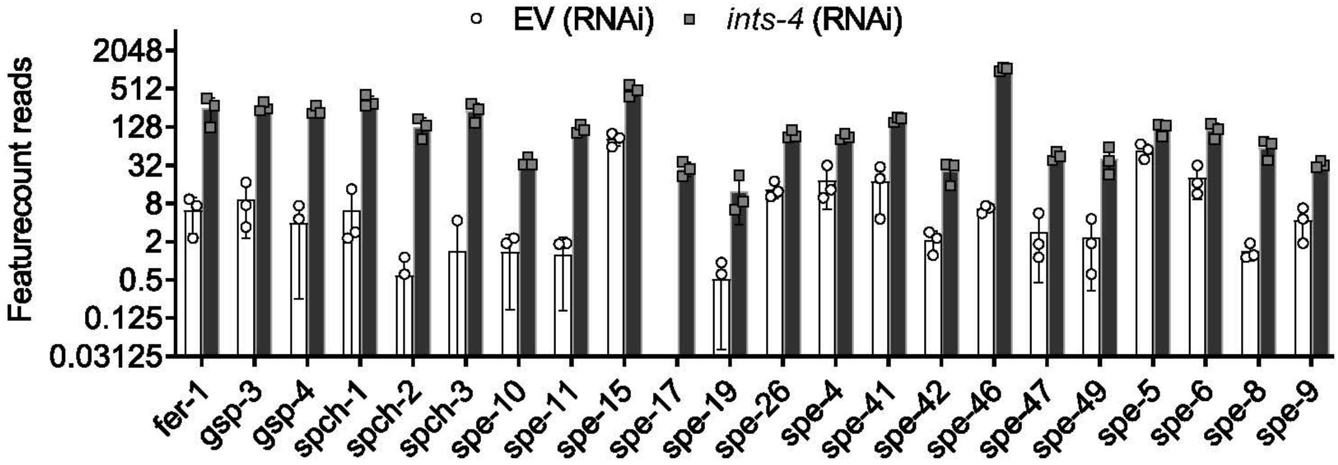

Fig. S1. Knockdown of *ints-4* increases reproductive gene expression. Featurecount reads of genes functioning in reproduction that was up-regulated by *ints-4* (RNAi) compared to EV (RNAi). Data obtained from Gómez-Orte et al., 2019.

Table S1. Lifespan assay statistics

Available for download at https://journals.biologists.com/bio/article/doi/10.1242/bio.061930/367336/Differential-effect-of-ubiquitous-and-germline#supplementary-data

Biology Open • Supplementary information
